# Interleukin-6 is a highly prognostic biomarker for POEMS syndrome

**DOI:** 10.1038/s41375-025-02659-7

**Published:** 2025-07-11

**Authors:** Joselle Cook, Rahma Warsame, Maryam Omar, Francis K. Buadi, Nadine Abdallah, David Dingli, Suzanne R. Hayman, Prashant Kapoor, Taxiarchis Kourelis, Moritz Binder, Eli Muchtar, Amie Fonder, Yi L. Hwa, Miriam A. Hobbs, Yi Lin, Wilson I. Gonsalves, Morie A. Gertz, Shaji K. Kumar, S. Vincent Rajkumar, Angela Dispenzieri

**Affiliations:** Division of Hematology, Department of Medicine, Mayo Clinic, Rochester, MN USA

**Keywords:** Haematological cancer, Myeloma, Disease-free survival

## To the Editor:

POEMS syndrome (Polyneuropathy, Organomegaly, Endocrinopathy, M-protein, and Skin changes) is a rare paraneoplastic clonal plasma cell disorder which remains a diagnostic challenge due to its diverse clinical manifestations [1]. POEMS can lead to interleukin-6 (IL-6) significant morbidity and multiorgan dysfunction [1]. With treatment, outcomes are favorable; after 2003 10-year overall survival (OS) was 79%, largely due to increased utilization of autologous stem cell transplant [1–3]. Elevated vascular endothelial growth factor (VEGF) levels correlate with POEMS disease activity and is used in response assessment [4]. VEGF’s role in angiogenesis and increasing vascular permeability may explain the organomegaly, skin changes, volume overload, and bone involvement present in POEMS syndrome [2]. The pathobiology is complex, involving multiple cytokines including interleukin-6 (IL-6) which upregulates VEGF production [5, 6]. However, the prognostic significance of IL-6 in POEMS remains unclear. We conducted a retrospective study to evaluate the significance of baseline IL-6 levels in newly diagnosed POEMS patients.

## Methods

We performed a retrospective analysis of patients with a new diagnosis of POEMS syndrome seen at Mayo Clinic, Rochester (2011 to 2019) who had IL-6 testing within 90 days of their diagnosis. Of the 117 patients seen during that period, 52 met inclusion criteria. Due to changes in the assay and different laboratories performing the IL-6 assay over the course of the study period, three different upper limits of normal (ULN) for IL-6 were applied based on the laboratory performing the analysis: <17.4 pg/mL from Viracor-IBT Laboratories (*n* = 3), <1.8 pg/mL from Mayo Clinic Laboratories (*n* = 5), and <5 pg/mL from Quest Diagnostics Nichols Institute, San Juan Capistrano (*n* = 44). IL-6 was classified as elevated if above the lab-specific ULN.

Hematologic, VEGF and positron emission tomography (PET) responses were assessed as previously described [2, 7]. Differences between groups were analyzed using Chi square. Because there are imperfect definitions for progression in POEMS syndrome, we calculated event-free survival (EFS), where an event was defined as disease progression, initiation of new therapy, or death. Planned treatment institution, such as  administering maintenance after ASCT, was not considered an event. EFS and OS were assessed using Kaplan-Meier analysis, with survival differences compared using the log-rank test. Univariate analysis evaluated prognostic impact of IL-6, albumin and the Wang score [3, 8]. *P* values less than 0.05 were considered significant. JMP (SAS) was used for statistical analysis. The study was approved by the Mayo Clinic Institutional Review board.

## Results

Among the 52 patients, 21 (40.4%) had elevated baseline IL-6 levels, (median value 3 times ULN; IQR 1.3, 4.4). The median (range) VEGF plasma levels across the cohort were 342.5 pg/ml (<31–2780) [normal range 31–86 pg/ml]. Elevated IL-6 was associated with male sex, hepatomegaly, ascites, cherry angiomata, low albumin, endocrinopathy, reduced Diffusing Capacity of the Lung for Carbon Monoxide (DLCO), mixed sclerotic/ lytic bone lesions, and higher light chain values (Table 1). IL-6 elevation was not associated with VEGF level (data not shown).

**Table 1:** Patient characteristics and responses to first-line therapies.

Characteristic	Normal Interleukin-6 *N* = 31	Elevated Interleukin-6^¶^ *N* = 21	*p* value
Male n (%)	20 (65)	19 (90)	0.03
Race/Ethnicity n(%)			0.051
White	27 (87)	15 (71)	
Black	4 (13)	2 (10)	
Pacific Islander	-	1 (5)	
Unknown	-	3 (14)	
Age, years, median (range)	52 (23-73)	59 (33-83)	0.09
Organomegaly* n (%)	14 (45)	15 (71)	0.06
Hepatomegaly n (%)	5 (16)	9 (43)	0.034
Splenomegaly n (%)	7 (23)	9 (43)	0.12
Lymphadenopathy n (%)	8 (26)	6 (29)	0.83
Castleman’s by biopsy n (%)	2 (6)	2 (10)	0.69
Endocrinopathy n (%)	27 (87)	21 (100)	0.037
Skin changes n (%)	26 (84)	15 (71)	0.3
Extravascular Fluid Overload n (%)	28 (90)	19 (90)	0.98
Peripheral edema	27 (87)	18 (86)	0.89
Effusions	6 (19)	8 (38)	0.13
Ascites	2 (6)	6 (29)	0.03
Erythrocytosis n (%)	6 (19)	2 (10)	0.32
Thrombocytosis n (%)	4 (13)	4 (19)	0.55
Abnormal DLCO (below 45% of predicted)* n (%)	1 (3)	5 (24)	0.02
Papilledema n (%)	4 (13)	2 (10)	0.71
Bone lesions n (%)	22 (71)	16 (76)	0.67
Sclerotic	18 (58)	14 (67)	0.53
Lytic	4 (13)	2 (10)	0.70
Mixed	9 (29)	12 (57)	0.04
None	9 (29)	5 (24)	0.68
Number of features present	6 (4-9)	6 (5-9)	0.19
VEGF pg/ml median (range)	435 (0-2780)	250 (45-816)	0.12
Plasma VEGF > 87 pg/mL n (%)	27 (87)	19 (90)	0.71
M-spike >0.5 g/dL n (%)	9 (60)	6 (40)	0.97
Kappa light chain mg/dl median (range)	1.7 (0.71-15.9)	3.73 (1.15-33.6)	0.008
Lambda light chain mg/dL median (range)	3.58 (0.879-11.8)	6.61 (1.48-21.2)	0.002
FLC-ratio median (range)	0.62 (0.132-1.96)	0.75 (0.139-2.15)	0.33
Aspartate Aminotransferase U/L median (range)	14 (7-40)	15 (6-127)	0.18
Alkaline phosphatase U/L median (range)	69 (42-282)	79 (31-432)	0.12
Total Bilirubin mg/dL median (range)	0.4 (0.2-1.4)	0.5 (0.2-1.7)	0.71
Direct Bilirubin mg/dL median (range)	0.1 (0.1-0.2)	0.1 (0.1-0.9)	0.81
Serum albumin, g/dL median (range)	3.45 (2.5-4.5)	3.1 (2.3-3.5)	0.0008
Bone marrow Plasma cell % median (range)	3(2-30)	5(2-20)	0.49
**Response to first line therapy**			
*Hematologic n(%)*			
CR	5(16)	3 (14)	*P* = 0.10**
VGPR	2(7)	3 (14)	
PR	0 (0)	2 (10)	
NR	19 (61)	7 (33)	
NE	5 (16)	6 (29)	
*VEGF n(%)*			
CR	16 (52)	10 (48)	*P* = 0.99**
PR	3 (10)	2 (10)	
NR	7 (22)	4 (19)	
NE	5 (16)	5 (23)	
*PET n(%)*			
CR	6 (24)	3 (21)	*P* = 0.95**
PR	13 (33)	8 (57)	
NR	6 (24)	3 (22)	

*CR* complete response, *DLCO* Diffusing Capacity of the Lung for Carbon Monoxide, *FLC* Free light chain, *IL-6* Interleukin-6 mg/dL milligrams per deciliter, *NR* no response, *NE* not evaluable or not evaluated, *PET* Positron Emission Tomography, *pg/mL* picograms per milliliter, *PR* partial response, *U/L* units per liter, *VGPR* very good partial response, *VEGF* Vascular Endothelial Growth Factor.

¶ Elevated IL-6 determined as elevated above the specific assay’s reference range.

*Organomegaly: as defined by radiologic report.

** P-value excludes the non-evaluable participants.

### Treatment

Upon the formal diagnosis of POEMS syndrome, most patients received systemic therapy including upfront high dose melphalan with autologous stem cell rescue (*n* = 18; 35%), lenalidomide or proteosome inhibitor based regimens (*n* = 14; 27%), non-transplant alkylator based therapy (cyclophosphamide) (*n* = 8; 15.%). One person with associated Castleman’s disease received IL-6 inhibitor, Siltuximab. Localized therapies were implemented in patients with limited bone lesions; radiation (*n* = 10; 19.2%) and cryoablation (*n* = 1; 1.9%).

We found that 29 patients were misdiagnosed as either chronic inflammatory demyelinating polyradiculoneuropathy (CIDP) or solitary plasmacytoma and received treatment prior to their formal diagnosis of POEMS syndrome and VEGF and IL-6 evaluations. The treatments administered prior to diagnosis of POEMS were steroids (*n* = 6, 21%) and intravenous immunoglobulin (*n* = 13, 46%) or immunosuppressive therapy such as mycophenolate mofetil, azathioprine or cyclophosphamide (*n* = 5, 18%). Others received radiation (*n* = 3, 11%) for presumed solitary plasmacytoma and one received low dose melphalan (*n* = 1, 4%) for possible but not confirmed POEMS. Baseline IL-6 levels did not differ significantly based on prior treatment (*p* = 0.12).

### Response assessment and survival

There was a trend for a higher likelihood of hematologic response (partial response or better) among those with elevated IL-6 (38% versus 23%, *p* = 0.09), but achievement of at least partial VEGF (PR_V_) and PET responses (PR_PET_) did not differ (*p* = 0.89, *p* = 0.85).

**Fig. 1:**
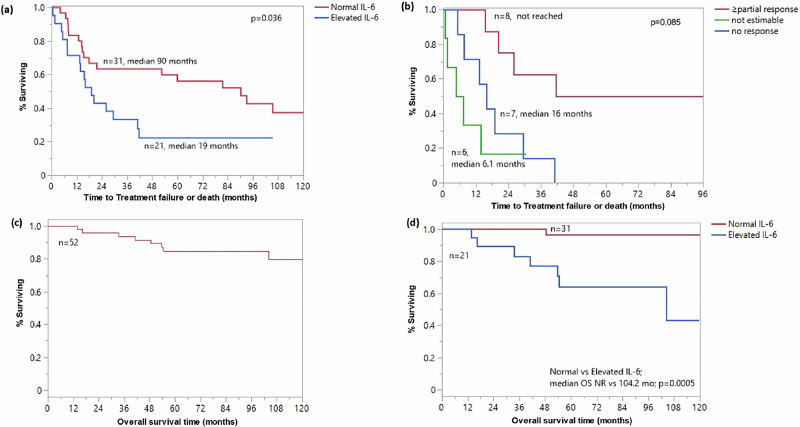
Interleukin-6 (IL-6) levels are associated with inferior failure-free and overall survival in patients with POEMS syndrome. **a** Event-free survival (EFS) stratified by baseline IL-6 level (normal vs. elevated); **b** EFS stratified by depth of hematologic response (≥partial response vs. no response) among patients with elevated IL-6; **c** overall survival (OS) for the full cohort; **d** OS stratified by baseline IL-6 level.

With median follow up of 83 months, 33 patients had events; 27 patients progressed and started a new therapy, while 6 patients died. The median EFS was 41 months (95%CI:16–93). Elevated IL-6 was associated with shorter median EFS (19 months, 95%CI:7.6–41 vs 90 months, 95%CI:17.9 to NR, *p* = 0.036) (Fig. 1a). Among those with elevated baseline IL-6 levels, lack of partial heme response (PR_H_) or better was associated with inferior median EFS (not reached versus 16 months versus 6.1 months for ≥ PR_H_, < PR_H_ and not evaluable, respectively, *p* = 0.085) (Fig. 1b). For patients with baseline elevated IL-6 levels, t here was no significant interaction between EFS and PR_V_ or PR_PET_ ( ≥ PR_V_ vs < PR_V_, 23.1 versus 13.3 months, *p* = 0.14 and ≥ PR_PET_ vs < PR_PET_, 29.4 vs 7.6 months; *p* = 0.36).

Median OS was not reached; 8-year OS was 85% (Fig. 1c). Elevated baseline IL-6 was associated with shorter OS (8-year OS of 64.4% and 97%, respectively, *p* = 0.005) (Fig. 1d) with a hazard ratio (HR) for death of 16.3 (95%CI 1.98-134.1; *p* = 0.0096).

 On univariate Cox proportional hazards analysis elevated IL-6, hypoalbuminemia, and a high Wang prognostic score were significantly associated with worse OS. Although confidence levels overlapped, IL-6 had the strongest effect, with a HR of 17.29 (95% CI: 2.1–142.3, *p* = 0.008), suggesting a markedly increased risk of mortality. Hypoalbuminemia was also a significant predictor, with an HR of 5.18 (95% CI: 1.28–20.8, *p* = 0.016), indicating that patients with lower albumin levels had poorer survival. Additionally, a high Wang prognostic score was associated with worse survival (HR 4.88, 95% CI: 1.21–19.6, *p* = 0.033), reinforcing its prognostic value. Given the small number of events, robust multivariate modeling to determine IL-6’s independence from other prognostic factors could not be performed.

## Discussion

We have demonstrated the clinical import of baseline IL-6 levels among patients with POEMS syndrome. Elevation of IL-6 is uniquely associated with specific presenting features, which are distinctly not associated with elevated VEGF, lymphadenopathy or coexisting Castleman disease. Among patients presenting with elevated IL-6, endocrinopathy, higher free light chains, lower serum albumin, ascites, hepatomegaly and abnormal DLCO were all more common. The role of IL-6 in driving inflammatory pathways is well described [9]. The ascites and hypoalbuminemia observed among patients with higher IL-6 likely reflect the effect of IL-6 on the liver, promoting acute phase reactants while decreasing albumin production [9]. Data suggest that in chronic inflammatory states, elevated IL-6 correlates with increased hepatocyte IL-6 expression and development of nonalcoholic steatohepatitis [10, 11]. It is therefore conceivable that the IL-6 itself drives hepatomegaly among POEMS patients with elevated IL-6. IL-6 is known to induce VEGF, so the lower VEGF levels among patients with elevated IL-6 remains unclear. We hypothesize that the elevation of multiple inflammatory pathways in POEMS syndrome together with high IL-6 may influence VEGF levels though this requires further evaluation in larger datasets or prospective studies [6].

Second, we have demonstrated that IL-6 is a highly prognostic biomarker. Patients with elevated baseline IL-6 had a much shorter event free survival than those without, especially among those patients who did not achieve a PR_H_ or better. This is certainly an important consideration in the patient evaluation and counseling and augurs for routine IL-6 level testing for all patients with POEMS.

Survival in POEMS is typically excellent with early treatment, but IL-6 levels at presentation remain a key prognostic differentiator. Patients with elevated IL-6 had a 17-fold increased risk of death compared to those with normal values. Elevated IL-6 may surpass the prognostic impact of hypoalbuminemia and complex risk scores (Wang et al.) though, larger studies are needed for multivariate validation [8].

Our findings are of great clinical consequence as IL-6 has not been routinely evaluated in POEMS due to unclear prognostic value - until now. Smaller studies have shown baseline IL-6/VEGF correlations and suggested IL-6 a disease burden biomarker [12]. A limitation of our study is the potential impact of pre-diagnostic treatments on baseline IL-6 measurements; some patients misdiagnosed as CIDP or solitary plasmacytoma received steroids or radiation before formal diagnosis. The small sample size, though expected for a rare disease limited advanced statistical analysis. Follow up IL-6 measurements were infrequent, preventing interpretation of longitudinal trends.

In summary, elevated IL-6 at diagnosis is associated with distinct clinical features, a shortened event free survival especially for those who do not achieve hematologic response, and perhaps most importantly, a strikingly inferior OS. IL-6 offers prognostic insight beyond hypoalbuminemia and the Wang score. This certainly warrants future study to examine IL-6 targeted strategies as an adjunct among such patients. In the meantime, our results establish that IL-6 is a clinically important biomarker among patients with POEMS syndrome.

## Data Availability

The data generated and analyzed during this study are available from the corresponding author upon reasonable request. Any proprietary or patient-identifiable data cannot be shared in accordance with institutional and ethical guidelines. For further details, please contact the corresponding author.
